# Essential Role for an M17 Leucine Aminopeptidase in Encystation of *Acanthamoeba castellanii*

**DOI:** 10.1371/journal.pone.0129884

**Published:** 2015-06-15

**Authors:** Yu-Ran Lee, Byoung-Kuk Na, Eun-Kyung Moon, Su-Min Song, So-Young Joo, Hyun-Hee Kong, Youn-Kyoung Goo, Dong-Il Chung, Yeonchul Hong

**Affiliations:** 1 Department of Parasitology and Tropical Medicine, Kyungpook National University School of Medicine, Daegu 700–422, Republic of Korea; 2 Department of Parasitology and Tropical Medicine, and Institute of Health Sciences, Gyeongsang National University School of Medicine, Jinju 660–751, Republic of Korea; 3 Department of Parasitology, Dong-A University, College of Medicine, Busan 602–714, Republic of Korea; Centro de Investigacion y de Estudios Avanzados del Instituto Politecnico Nacional, MEXICO

## Abstract

Encystation of *Acanthamoeba* leads to the formation of resilient cysts from vegetative trophozoites. This process is essential for parasite survival under unfavorable conditions such as starvation, low temperatures, and exposure to biocides. During encystation, a massive turnover of intracellular components occurs, and a large number of organelles and proteins are degraded by proteases. Previous studies with specific protease inhibitors have shown that cysteine and serine proteases are involved in encystation of *Acanthamoeba*, but little is known about the role of metalloproteases in this process. Here, we have biochemically characterized an M17 leucine aminopeptidase of *Acanthamoeba castellanii* (AcLAP) and analyzed its functional involvement in encystation of the parasite. Recombinant AcLAP shared biochemical properties such as optimal pH, requirement of divalent metal ions for activity, substrate specificity for Leu, and inhibition profile by aminopeptidase inhibitors and metal chelators with other characterized M17 family LAPs. AcLAP was highly expressed at a late stage of encystation and mainly localized in the cytoplasm of *A*. *castellanii*. Knockdown of AcLAP using small interfering RNA induced a decrease of LAP activity during encystation, a reduction of mature cyst formation, and the formation of abnormal cyst walls. In summary, these results indicate that AcLAP is a typical M17 family enzyme that plays an essential role during encystation of *Acanthamoeba*.

## Introduction

*Acanthamoeba* spp. are causative agents of granulomatous amoebic encephalitis and amoebic keratitis. The life cycle of *Acanthamoeba* consists of two stages, the vegetative trophozoite and the dormant cyst. Under challenging conditions such as starvation, low temperatures, and exposure to biocides, the trophozoite converts to the resilient cyst form [1–3]. *Acanthamoeba* cysts have double walls; the inner cyst wall (endocyst) is partially composed of cellulose, and the outer cyst wall (ectocyst) consists of acid-insoluble protein-containing materials [4, 5]. This differentiation, termed encystation, protects *Acanthamoeba* against host immune responses and allows it to evade the effects of disinfectants and chemotherapeutic agents owing to the high resistance of the cysts (reviewed in [3]). Therefore, inhibition of encystation during the treatment of amoebic infections could lead to more favorable outcomes. However, realizing this goal is hindered by a lack of information about the encystation mechanism.

Proteolysis, through the lysosomal (autophagic) pathway or the ubiquitin-proteasome pathway, plays an important role in many biological processes, including differentiation. Many proteases such as cysteine, serine, and metal proteases are utilized for encystation and excystation (reversion to trophozoites) of protozoan parasites (reviewed in [6]). The cysteine protease GICP2 [7, 8] and the subtilisin-like proprotein convertase gSPC [9] have been implicated in encystation and excystation of *Giardia lamblia*. *Giardia* dipeptidyl peptidase IV, a membrane-associated aminopeptidase, was identified as a target of bestatin and may play a role in encystation [10]. Bestatin abolished the expression of cyst wall protein and blocked cyst formation without affecting the viability of *G*. *lamblia* trophozoites [10]. In *Entamoeba invadens*, autophagy and the ubiquitin-proteasome pathway were shown to be involved in differentiation [11–13], and cysteine protease inhibitors reduced encystation efficiency [14, 15].

Previous studies have shown that phenylmethylsulfonyl fluoride (PMSF), a serine protease inhibitor, and ethylenediaminetetraacetic acid (EDTA), a metal chelating agent, markedly diminish cyst formation and excystation efficiency in *Acanthamoeba castellanii* [16, 17], suggesting that serine proteases and metalloproteases or metal ions play important roles in *Acanthamoeba* differentiation. We have previously found that during encystation of *Acanthamoeba*, large numbers of organelles and proteins are degraded by an encystation-mediating serine protease [18]. However, the only two extracellular metalloproteases identified so far (molecular weights of 150 kDa and 80 kDa, respectively) fulfill other tasks. The former degrades extracellular components, and its activity is inhibited by 1,10-phenanthroline [19, 20], while the latter has its expression and secretion induced by mannose-mediated adhesion of *Acanthamoeba* to host cells [21]. To date, no functional metalloprotease has been implicated in encystation of *Acanthamoeba*.

In a previous microarray study, we found a large increase in the expression level of leucine aminopeptidase during encystation of *A*. *castellanii* [22]. Leucine aminopeptidase (EC 3.4.11.1; LAP), a member of the M17 family, is a metalloexopeptidase localized in the cytoplasm of eukaryotes and is presumably involved in the processing and regular turnover of intracellular proteins [23]. LAP catalyzes the removal of N-terminal amino acids from peptides in which the N-terminal residue is preferably Leu but may be any amino acid, except for Arg or Lys. It possesses two metal ions essential for catalysis, and its activity is inhibited by bestatin, amastatin, and metal chelators [23, 24]. The LAPs of protozoan parasites such as *Toxoplasma*, *Cryptosporidium*, *Trypanosoma*, *Leishmania*, and *Plasmodium* have been shown to be involved in free amino acid regulation [25–29] and are considered chemotherapeutic drug targets or vaccine candidates because of their important roles in parasite biology. However, little is known about the presence of LAPs in *Acanthamoeba* or their involvement in differentiation.

Here, we identified and characterized an M17 leucine aminopeptidase of *A*. *castellanii* (AcLAP). The enzyme was highly expressed at the encystation stage, and its knockdown interrupted the encystation process of *A*. *castellanii*, indicating an important role for this enzyme in the encystation of *Acanthamoeba*.

## Materials and Methods

### Cultivation of amoebae and induction of encystation

*A*. *castellanii* Castellani of the T4 genotype, which was originally isolated as a eukaryotic cell culture contaminant, was obtained from the American Type Culture Collection (ATCC #30011; Manassas, VA, USA). Amoebae were cultured axenically in peptone-yeast-glucose (PYG) medium at 25°C. Encystment was induced as previously described with slight modifications [30]. Briefly, approximately 5 × 10^5^ cells in the post-logarithmic growth phase were collected aseptically, washed with phosphate-buffered saline (PBS) and incubated in 10 mL of encystation medium (95 mM NaCl, 5 mM KCl, 8 mM MgSO_4_, 0.4 mM CaCl_2_, 1 mM NaHCO_3_, 20 mM Tris-HCl, pH 9.0) for 72 h. The morphological change of cells to cysts was observed under a light microscope. Encystation efficiency was calculated by counting cysts after treating cells with 0.05% sarkosyl and 0.22% trypan blue, which selectively stains nonviable cells [11, 31].

### Cloning of the AcLAP gene and preparation of recombinant AcLAP (AcLAPr) and antiserum against AcLAPr

The full-length cDNA sequence of *A*. *castellanii* AcLAP (cluster ID ACL00003969) was isolated from the Taxonomically Broad EST Database (http://tbestdb.bcm.umontreal.ca) and verified by reverse transcription-polymerase chain reaction. The deduced amino acid sequences were aligned using ClustalW (Geneious Pro 5.5.7; http://www.geneious.com). The phylogenetic tree was constructed using the neighbor-joining method with MEGA 6 software (http://www.megasoftware.net). Bootstrap proportions were used to assess the robustness of the tree with 1000 bootstrap replications. For the production of recombinant AcLAP (AcLAPr) protein, the PCR product amplified from cDNA using the primers sense 5′-ATTGAATTCATGCAGGGTAAGGCGAAGTGCT-3′ and antisense 5′-AATTCTAGACTAGTGCTTCTCCACCTTGT-3′ was digested with *Eco*RI and *Xba*I and cloned into the pCold TF vector (Takara Bio, Tokyo, Japan) to express AcLAP fused to a trigger factor (TF), a prokaryotic ribosome-associated chaperone protein that facilitates co-translational folding of nascent polypeptides. The TF tag facilitated the expression of soluble AcLAPr by promoting correct protein folding. *Escherichia coli* BL21 (pKJE7) cells (Novagen, San Diego, CA, USA) transformed with pCold-TF-AcLAP were cultured in Luria-Bertani broth supplemented with 100 μg/mL ampicillin, grown at 37°C, and induced with isopropyl-β-d-thiogalactoside at a final concentration of 0.1 mM for 20 h at 15°C. The cells were harvested, lysed via sonication for 5 min with a 15 s on/15 s off cycle (Branson, Danbury, CT, USA), and centrifuged. Then, His_6_-tagged TF-fused AcLAPr was purified from the soluble fraction of *E*. *coli* cells with nickel-nitrilotriacetic acid (Ni-NTA) agarose (Qiagen, Valencia, CA, USA) according to the manufacturer’s instructions. The N-terminal His_6_-tagged TF tag was removed using a thrombin cleavage capture kit (Novagen) according to instructions supplied with the kit. Protein concentration was determined via Bradford assay (Bio-Rad, Hercules, CA, USA), and purity was assesses by sodium dodecyl sulfate–polyacrylamide gel electrophoresis (SDS-PAGE) with Coomassie Brilliant Blue staining (S1 Fig).

The rat antiserum specific for AcLAPr (anti-AcLAPr) was produced by immunizing rats with purified AcLAPr. Briefly, purified AcLAPr (>100 μg) was subcutaneously injected into two 6-week-old Sprague-Dawley rats (Bionics, Seoul, South Korea) with an equal volume of Freund’s complete adjuvant (Sigma-Aldrich). Before immunization, negative blood samples were collected from the rats for control experiments. Two AcLAPr boosters were administered 14 and 28 days after the first injection and contained AcLAPr (>100 μg) in equal volumes of Freund’s incomplete adjuvant (Sigma-Aldrich). Blood samples were collected from the rats before booster administration to monitor antibody production. Antiserum was obtained from the heart 14 days after the final booster. Anti-AcLAPr antibodies were obtained from the blood via centrifugation for 15 min at 4°C and analyzed with western blotting.

For immunoblot assays, cell lysates of *A*. *castellanii* trophozoites and cysts (24, 48, or 72 h after encystation induction) were separated with SDS-PAGE. Proteins were transferred from the gels to polyvinylidene fluoride membranes, which were then blocked overnight in 5% skim milk in Tris-buffered saline at 4°C, washed with Tris-buffered saline containing 0.05% Tween 20 (TBST), and incubated with 1:1,000 diluted AcLAPr antiserum for 2 h at room temperature. After washing with TBST, the membranes were incubated with 1:2,000 diluted rat immunoglobulins/horseradish peroxidase-labeled antibodies (Dako, Glostrup, Denmark) for 30 min at room temperature. Proteins were detected with chemiluminescence using enhanced chemiluminescence western blotting detection reagents (Amersham Biosciences, Piscataway, NJ, USA). Membranes were stripped, reprobed with mouse monoclonal anti-actin antibody (1:5,000 dilution; Santa Cruz Biotechnology, Santa Cruz, CA, USA) for 2 h and cross-reacted with *Acanthamoeba* actin [32] to control for equal loading. Membranes were washed with TBST and incubated with 1:2,000 diluted mouse immunoglobulins/horseradish peroxidase-labeled antibodies (Dako) for 30 min at room temperature. The band intensities of western blots were quantified with a gel documentation system and Quantity One software (Bio-Rad, Hercules, CA, USA).

### Biochemical properties of AcLAPr

The aminopeptidase activity of AcLAPr was assayed fluorometrically as the hydrolysis of l-leucine-7-amido-4-methylcoumarin hydrochloride (Leu-AMC) (Sigma-Aldrich). Briefly, 10 μL of enzyme solution was added to 190 μL of assay buffer (50 mM Tris-HCl, 1 mM MnCl_2_, pH 8.0) containing 10 μM Leu-AMC and incubated for 20 min at 37°C, and the release of fluorescence was measured at an excitation wavelength of 370 nm and an emission wavelength of 440 nm using a Gemini EM fluorescence microplate reader (Molecular Devices, Sunnyvale, CA, USA). The optimal pH for AcLAPr activity was determined in sodium phosphate buffer (pH 4.0–6.0), Tris-HCl buffer (pH 7.0–9.0), and glycine-NaOH buffer (pH 10.0–12.0). AcLAPr was added to each buffer, and enzyme activity was measured as described above. To determine the effect of metal ions on AcLAPr activity, AcLAPr was preincubated with different concentrations of metal chlorides (CaCl_2_, CoCl_2_, MgCl_2_, MnCl_2_, NiCl_2_, or ZnCl_2_) in 50 mM Tris-HCl buffer (pH 8.0) for 20 min at 37°C. The effects of an aminopeptidase inhibitor (bestatin; Sigma-Aldrich) and metal chelators (EDTA and 1,10-phenanthroline; Sigma-Aldrich) on AcLAPr activity were assessed by preincubating the enzyme with different concentrations of the reagents in 50 mM Tris-HCl buffer (pH 8.0) containing 1 mM MnCl_2_ for 20 min at 37°C, after which Leu-AMC (10 μM) was added and activity assayed as described above. The substrate specificity of AcLAPr was determined using Leu-AMC, l-arginine-AMC (Arg-AMC), and l-methionine-AMC (Met-AMC) (all from Sigma-Aldrich) as substrates. All assays were carried out in triplicate, and the means and standard deviations were calculated. The kinetic parameters of AcLAPr for each substrate were determined using a constant amount of enzyme (25 nM) and varying concentrations of substrate (0–100 mM).

### Transient transfection

To determine the intracellular localization of AcLAP, the AcLAP gene was cloned into the pGAPDH vector, which contains an *Acanthamoeba* glyceraldehyde 3-phosphate dehydrogenase (GAPDH) promoter for constitutive expression and an enhanced green fluorescent protein (EGFP) reporter gene [33]. The AcLAP gene was PCR-amplified with primers that incorporated an *Nde*I restriction site at the 5′-end (5′-ATTCATATGATGCAGGGTAAGGCGAAGTGC-3′) and an *Spe*I restriction site at the 3′-end (5′-AATACTAGTGTGCTTCTCCACCTTGTCCCG-3′) and inserted into the pGAPDH vector upstream of the EGFP gene (pGAPDH-AcLAP-EGFP). The amplified DNA fragment was sequenced completely (Macrogen, Seoul, Korea). *A*. *castellanii* was transfected with the pGAPDH-AcLAP-EGFP plasmid as previously described [18]. Briefly, approximately 4 × 10^5^ cells/well were seeded into a six-well culture plate in 3 mL PYG medium and incubated overnight at 25°C. Plasmid DNA (4 μg) in 100 μL amoeba culture medium was mixed with 20 μL SuperFect transfection reagent (Qiagen), incubated 10 min at room temperature, and added to the cells. After incubation at 25°C for 3 h to allow for uptake of DNA-SuperFect complexes, the cells were washed with PBS, incubated for 24 h, and placed in encystation medium and incubated for 24, 48, or 72 h. AcLAP-EGFP expression was assessed with fluorescence microscopy.

### Knockdown of AcLAP

Small interfering RNA (siRNA) targeting the AcLAP gene of *A*. *castellanii* was synthesized by Sigma-Proligo (Boulder, CO, USA) based on the cDNA sequence of the gene. The sequence of the upper strand was 5′-CUCAGAAGGUUGAGGAGAA-3′. The siRNA oligonucleotides were labeled with fluorescein isothiocyanate to allow transfection efficiency to be determined by flow cytometry (FACStar flow cytometer; BD Biosciences, Mountain View, CA, USA). *A*. *castellanii* trophozoites plated at a density of 4 × 10^5^ cells/well were transfected with siRNA (4 μg) against AcLAP or with scrambled siRNA as a negative control (Ambion, Austin, TX, USA) following the methodology described above for the transient transfection assay of pGAPDH-AcLAP-EGFP, and the transfection efficiencies were determined. After incubation for 24 h at 25°C, the transfected cells were placed into encystation medium and incubated for 24, 48, or 72 h. Then, total RNA from scrambled siRNA- and AcLAP siRNA-transfected cells was purified with an RNeasy Mini kit (Qiagen), and the first strand of cDNA was synthesized from 1 μg total RNA from each sample with a RevertAid first-strand cDNA synthesis kit (Fermentas, Hanover, IN, USA) according to the manufacturer’s instructions. The expression levels of AcLAP in scrambled siRNA- and AcLAP siRNA-transfected cells were examined with quantitative real-time PCR (qRT-PCR) as previously described [32]. Briefly, the PCR reactions were carried out using 1 μL of the synthesized cDNAs as template in a 20 μL volume containing 10 μL of Power SYBR Green PCR Master Mix (PE Applied Biosystems, Foster, CA, USA), 0.2 μL of 10 pmol/μL AcLAP sense primer (5′-AAGACCAAGGCGATCTGTCTCA-3′), 0.2 μL of 10 pmol/μL AcLAP antisense primer (5′-CCTCAACCGTCTTGCCATTCT-3′), and 8.6 μL of distilled water. Relative amounts were calculated and normalized with respect to the level of the internal standard, *Acanthamoeba* actin (GenBank accession no. CAA23399) [34] (sense primer 5′-AGGTCATCACCATCGGTAACG-3′ and antisense primer 5′-TCGCACTTCATGATCGAGTTG-3′). qRT-PCR was performed with GeneAmp 5700 Sequence Detection System (PE Applied Biosystems) using the default thermocycler program for all genes: 10 min of preincubation at 95°C followed by 40 cycles of 15 s at 95°C and 1 min at 60°C.

LAP activities in extracts of cells transfected with siRNA against AcLAP or scrambled siRNA were determined using Leu-AMC. Cell extracts (10 μg) were preincubated in reaction buffer (1 mM MnCl_2,_ 50 mM Tris-HCl, pH 8.0) at 37°C for 30 min before adding 10 μM Leu-AMC. After incubation at 37°C for 1 h, reactions were monitored as described above. The effect of an aminopeptidase inhibitor and metal chelators on the encystation of *Acanthamoeba* were assessed by counting of mature cysts as described above after incubation with different concentration of reagents. Statistical analysis was performed using the SPSS statistics program (version 19; IBM, New York, NY). Triplicate experiments were performed and the means, standard deviation, and standard error means were calculated for statistical comparisons. Mean differences were examined by Student's t-test (two-tailed) or by ANOVA. Significance was taken at a value of *P* < 0.05.

### Fluorescence microscopy and transmission electron microscopy

Amoebae expressing AcLAP-EGFP were isolated by flow cytometry (FACSAria cell sorter, BD Biosciences), washed with PBS, and incubated with 50 nM LysoTracker Red DND 99 (Molecular Probes, Eugene, OR, USA) for 1 h at room temperature. After being washed again with PBS, the cells were transferred to glass-bottom microwell dishes (MatTek, Ashland, MA, USA) and incubated for 30 min at 25°C to allow adhesion to the dishes. Cells were examined by confocal laser scanning microscopy using an LSM 5 EXCITER scalable confocal microscope (Carl Zeiss, Hamburg, Germany). AcLAP-EGFP and LysoTracker Red were measured using band-pass filters that provided excitation and emission wavelengths of 500–530 nm and 570–590 nm, respectively. For transmission electron microscopy (TEM), cell suspensions were centrifuged, sediments were washed three times in cold PBS, pre-fixed with 2.5% glutaraldehyde in 0.1 M phosphate buffer (pH 7.4) for 3 h, rinsed with 0.1 M phosphate buffer, post-fixed with 1% osmium tetroxide for 2 h, rinsed twice with 0.1 M phosphate buffer, dehydrated using an ethyl alcohol gradient (50%, 70%, 80%, 95%, and 100%), treated twice with propylene oxide resin (1:1 dilution) for 20 min, embedded in epoxy resin (EMbed-812; Electron Microscopy Sciences, Hatfield, PA, USA), and incubated at 37°C for 12 h, 45°C for 12 h, and 60°C overnight. Ultrathin sections were cut on a Reichert-Jung ultramicrotome, stained with uranyl acetate and lead citrate, and analyzed using a model H-7000 TEM (Hitachi, Tokyo, Japan).

## Results

### Identification of the AcLAP gene

**Fig 1:**
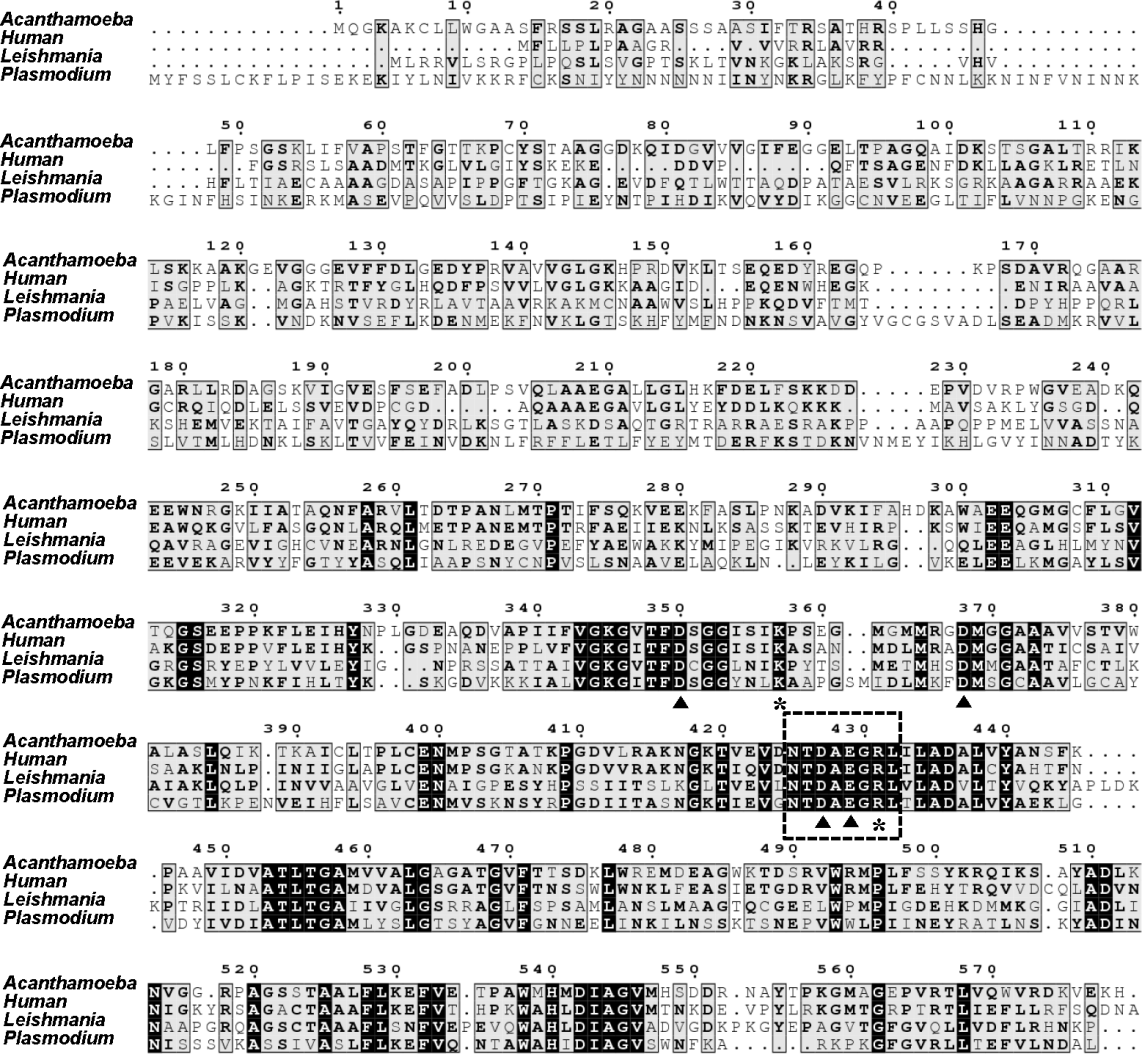
Identification of AcLAP as an M17 aminopeptidase of *Acanthamoeba castellanii*. Multiple alignment of the deduced amino acid sequence of AcLAP with leucine aminopeptidase (LAP) sequences of human (GenBank accession no. CAG33409) (48% amino acid identity), *Leishmania major* (GenBank accession no. XP_001683430) (58% amino acid identity), and *Plasmodium falciparum* (GenBank accession no. AAN37052) (66% amino acid identity) was performed using ClustalW software. Black and gray boxes indicate identical and conserved amino acids, respectively. The M17 aminopeptidase family signature sequence, NTDAEGRL, is indicated by a box with a dashed border. Metal-coordinating residues and active-site residues are indicated by closed triangles and asterisks, respectively.

**Fig 2:**
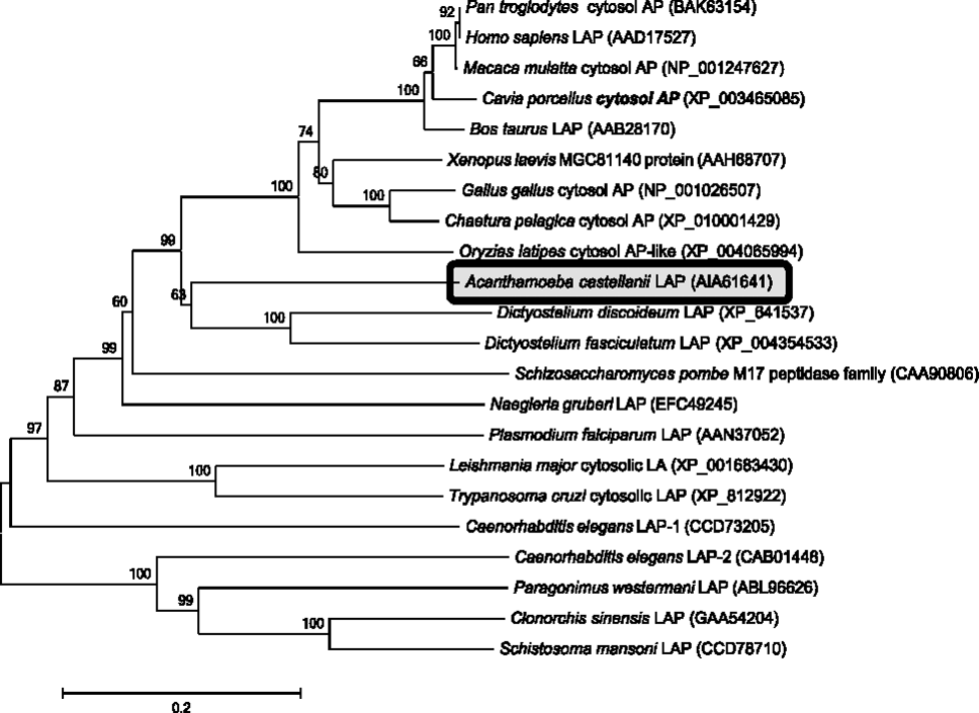
Phylogenetic analysis of AcLAP. The phylogenetic tree was constructed with the neighbor-joining method using the MEGA 6 software. Numbers on the branches indicate bootstrap proportions (1000 replicates).

A previous microarray study of the cyst-specific gene expression profile of *Acanthamoeba* showed that LAP-like proteins are up-regulated during encystation [22]. As the functional role of LAP in encystation has not been studied in *Acanthamoeba*, we cloned the cDNA of a LAP-like protein. The gene corresponding to LAP contained a 1740-bp open reading frame encoding a protein of 579 amino acids with a calculated molecular weight of 61.8 kDa. When the deduced amino acid sequence was aligned with other related protein sequences, it showed high sequence similarity with M17 aminopeptidases [35] characterized in other organisms such as *Plasmodium falciparum* [28], *Leishmania major* [24], and human [36] (Fig 1). The encoded protein was found to contain the highly conserved M17 aminopeptidase family signature sequence (NTDAEGRL, residues 425–432) [37] and highly conserved amino acid residues for metal binding (D350, D368, D427, E429) and catalytic site formation (K357, R431) (Fig 1). Therefore, this sequence was designated AcLAP (*Acanthamoeba castellanii* leucine aminopeptidase; GenBank accession no. KJ572802). Phylogenetic analysis revealed that AcLAP clustered into a clade closely related with LAPs from Amoebozoa, including *Dictyostelium* sp. (Fig 2).

### AcLAP is a functional enzyme highly expressed during encystation

**Fig 3:**
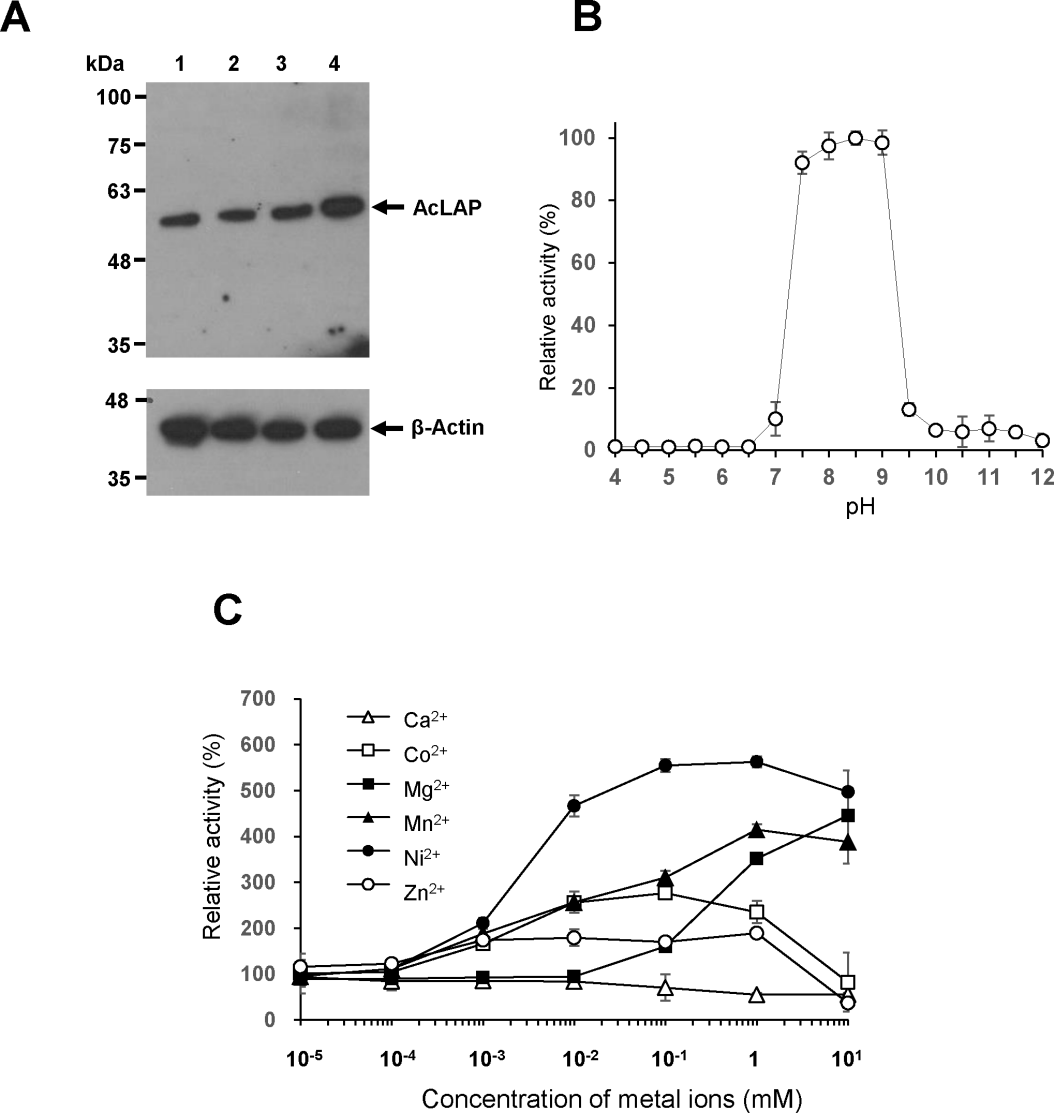
Functional and biochemical analysis of AcLAP. (A) Change in expression of AcLAP during encystation of *A*. *castellanii*. Crude trophozoite extracts (lane 1) and cyst extracts at 24 h (lane 2), 48 h (lane 3), and 72 h (lane 4) after induction of encystation of *A*. *castellanii* were resolved by sodium dodecyl sulfate-polyacrylamide gel electrophoresis, and western blotted with rat polyclonal anti-AcLAPr antibody (upper panel); proteins were stripped and reprobed with anti-actin antibody to ensure equal loading (lower panel). (B) Optimum pH of AcLAPr activity assayed at 37°C for 1 h with Leu-AMC substrate as described in the materials and methods section. (C) Influence of divalent metal cations on AcLAPr activity. Data represent the mean relative activities ± SD of triplicate measurements.

We examined expression levels of AcLAP in *A*. *castellanii* trophozoites and cysts at 24 h, 48 h, and 72 h after induction of encystation by western blot using an anti-AcLAPr polyclonal antibody. As shown in Fig 3A, expression of AcLAP was detected in trophozoites (lane 1), with no significant change of its level at 24 h and 48 h, yet an approximate twofold increase was observed at 72 h post-encystation (lanes 2–4).

To ascertain the enzymatic characteristics of AcLAP, we produced recombinant AcLAP (AcLAPr) and characterized its biochemical properties. AcLAPr showed aminopeptidase activity in a broad optimal pH range (pH 7.5–9.0), with a maximum at pH 8.5 (Fig 3B). Since leucine aminopeptidases of the M17 family are metalloenzymes, metal cations are required for the maintenance of their enzymatic activity and stability [38]. Therefore, we examined the effects of divalent metal cations on AcLAPr activity. As shown in Fig 3C, AcLAPr activity was progressively enhanced by Ni^2+^, Mn^2+^, Mg^2+^, Zn^2+^, or Co^2+^ but not by Ca^2+^. Among the divalent metal ions tested, Ni^2+^ (100 μM to 1 mM) increased AcLAPr activity the most (5.5-fold). However, higher concentrations of all metal ions (1–10 mM) except Mg^2+^ inhibited AcLAPr activity. We studied the inhibition of AcLAPr using metal chelating reagents or the specific aminopeptidase inhibitor bestatin. The metal chelators EDTA and 1,10-phenanthroline abolished AcLAPr activity at concentrations of 1 mM and 10 mM, respectively (Table 1).

**Table 1:** Effects of metal chelators and bestatin on the activity of AcLAPr.

	Concentration	Activity (%)
**Control**		100
**Metal chelator**		
**EDTA**	10 μM	94.16 ± 0.60
	100 μM	93.64 ± 0.54
	1 mM	1.76 ± 0.05
**1,10-Phenanthroline**	0.1 mM	92.35 ± 1.16
	1 mM	92.06 ± 0.40
	10 mM	2.24 ± 0.03
**Aminopeptidase inhibitor**		
**Bestatin**	1 μM	96.69 ± 0.59
	2 μM	83.95 ± 1.73
	4 μM	54.90 ± 1.96
	10 μM	22.54 ± 1.13

Bestatin proved to be an inhibitor of AcLAPr activity too, with only 23% of the enzymatic activity remaining after preincubation with 10 μM bestatin (Table 1). Regarding substrate specificity, AcLAPr readily hydrolyzed Leu-AMC and exhibited substantial amidolytic activity against Met-AMC but showed little activity against Arg-AMC (Table 2).

**Table 2:** Kinetic parameters for AcLAPr.

Substrate	*k*cat (s^-1^)	*K*m (μM)	*k*cat/*K*m (s^-1^ M^-1^)
**Arg-AMC**	0.13 ± 0.03	41.37 ± 0.05	3.14 × 10^2^
**Leu-AMC**	0.83 ± 0.11	13.51 ± 1.31	6.14 × 10^3^
**Met-AMC**	0.33 ± 0.04	3.77 ± 0.46	8.49 × 10^3^

*k*cat, turnover number; *K*m, Michaelis-Menten constant.

### Encystation is suppressed in AcLAP knockdown cells

**Fig 4:**
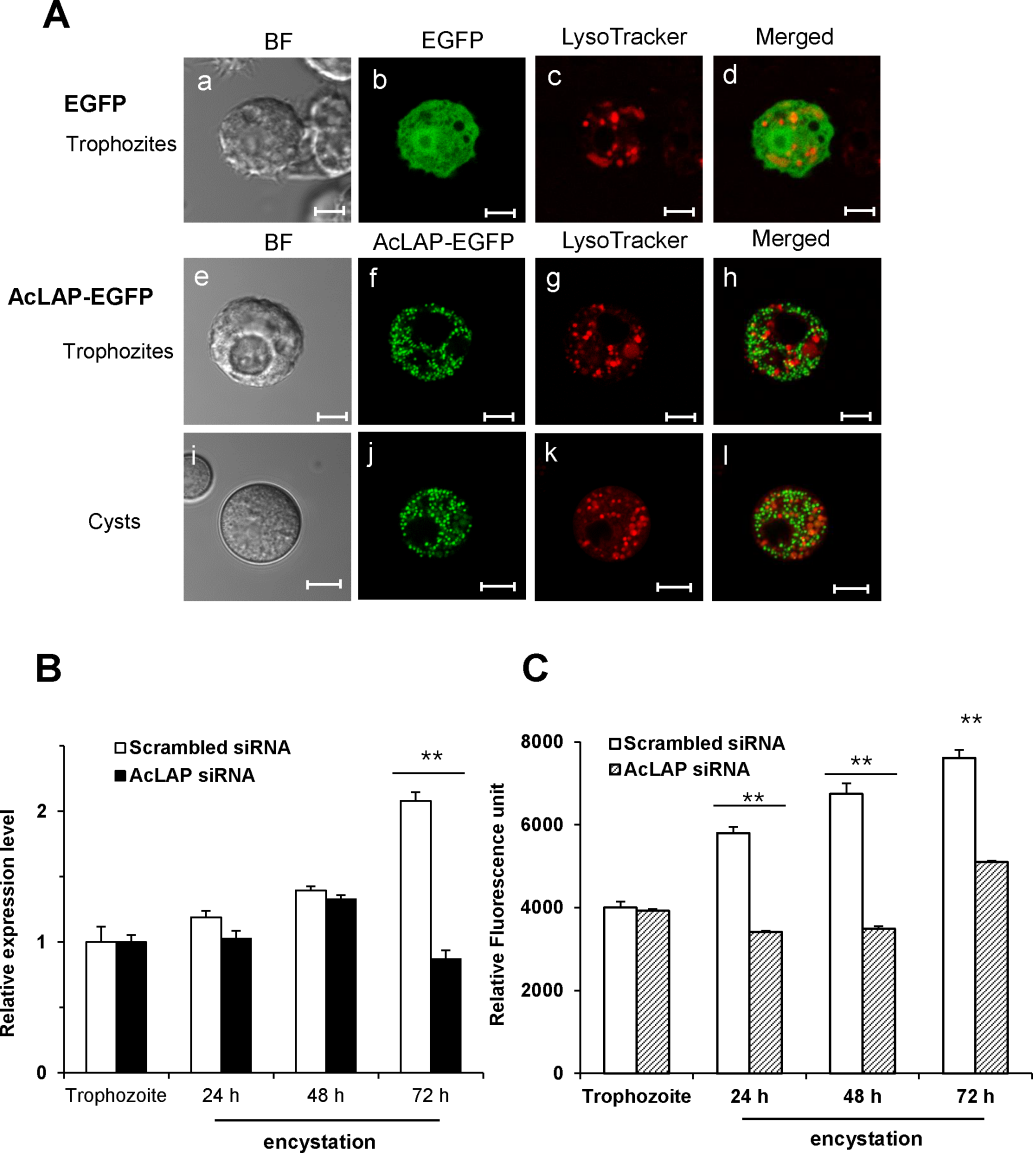
Intracellular localization of AcLAP and inhibition of encystation by siRNA-mediated silencing of AcLAP. (A) Trophozoites (a-h) transfected with pGAPDH-EGFP or pGAPDH-AcLAP-EGFP (green), transferred into a medium to induce encystation, incubated for 72 h (i-l), and examined under a fluorescence microscope. Lysosomes and autophagolysosomes were visualized by LysoTracker Red staining (red), and the resulting merged images are shown (yellow). BF denotes bright-field images. Bar = 5 μm. (B) Trophozoites transfected with scrambled siRNA (light bars) or AcLAP siRNA (dark bars), transferred into encystation medium, incubated for 24 h, 48 h, or 72 h, and examined for transcriptional changes of AcLAP with quantitative real-time polymerase chain reaction. Transfection efficiencies (79 to 85%) of FITC-conjugated AcLAP siRNA were determined by fluorescence-activated cell sorting analysis. The expression of AcLAP was normalized to that of *Acanthamoeba* actin. (C) LAP activity of *A*. *castellanii* cysts. Extracts from cells transfected with scrambled siRNA (open bars) or AcLAP siRNA (patterned bars) were prepared 1 (corresponding to trophozoite), 24, 48, and 72 h post-encystation. LAP activity was measured using Leu-AMC as a substrate. Data represent the means ± SD of experiments performed in triplicate; double asterisk, *P* < 0.01.

To determine the intracellular localization of AcLAP, *A*. *castellanii* trophozoites were transfected with the plasmid containing the EGFP (pGAPDH-EGFP) or EGFP-fused AcLAP gene (pGAPDH-AcLAP-EGFP) (Fig 4A), transferred to the encystation medium, and examined by fluorescence microscopy following staining with LysoTracker Red, which labels acidic compartments such as lysosomes and autophagolysosomes [39, 40]. As shown in Fig 4A, the distribution of AcLAP-EGFP fusion protein in trophozoites and cysts showed multiple small vesicle-like structures in the cytoplasm of both *A*. *castellanii* trophozoites and cysts, whereas no colocalization was observed with the LysoTracker-labeled compartments.

To examine whether AcLAP is involved in encystation of *Acanthamoeba*, siRNA-mediated gene silencing was used to reduce the expression level of endogenous AcLAP. After transfection with siRNA against AcLAP or scrambled control siRNA, trophozoites were transferred into encystation medium, and the expression level of AcLAP was examined by qRT-PCR. As shown in Fig 4B, AcLAP mRNA expression in cysts at 72 h after encystation induction was decreased to 40% of the AcLAP transcriptional level in scrambled-siRNA transfectants (*P* < 0.01). Then, to examine whether siRNA-mediated silencing of AcLAP expression affects AcLAP activity during encystation, we analyzed AcLAP activity in scrambled siRNA- and AcLAP siRNA-transfected cells with Leu-AMC as a substrate. This analysis confirmed the effect of siRNA-mediated silencing of AcLAP in *A*. *castellanii* cysts. LAP activity gradually increased at 24 h, 48 h, and 72 h post-encystation in scrambled siRNA-transfected cells, indicating an increase of LAP activity during the encystation process (*P* < 0.01) (Fig 4C). Meanwhile, LAP activity decreased in AcLAP siRNA-transfected cells compared to scrambled siRNA-transfected cells, suggesting AcLAP siRNA effectively inhibited the expression of AcLAP in the transfected cells.

**Fig 5:**
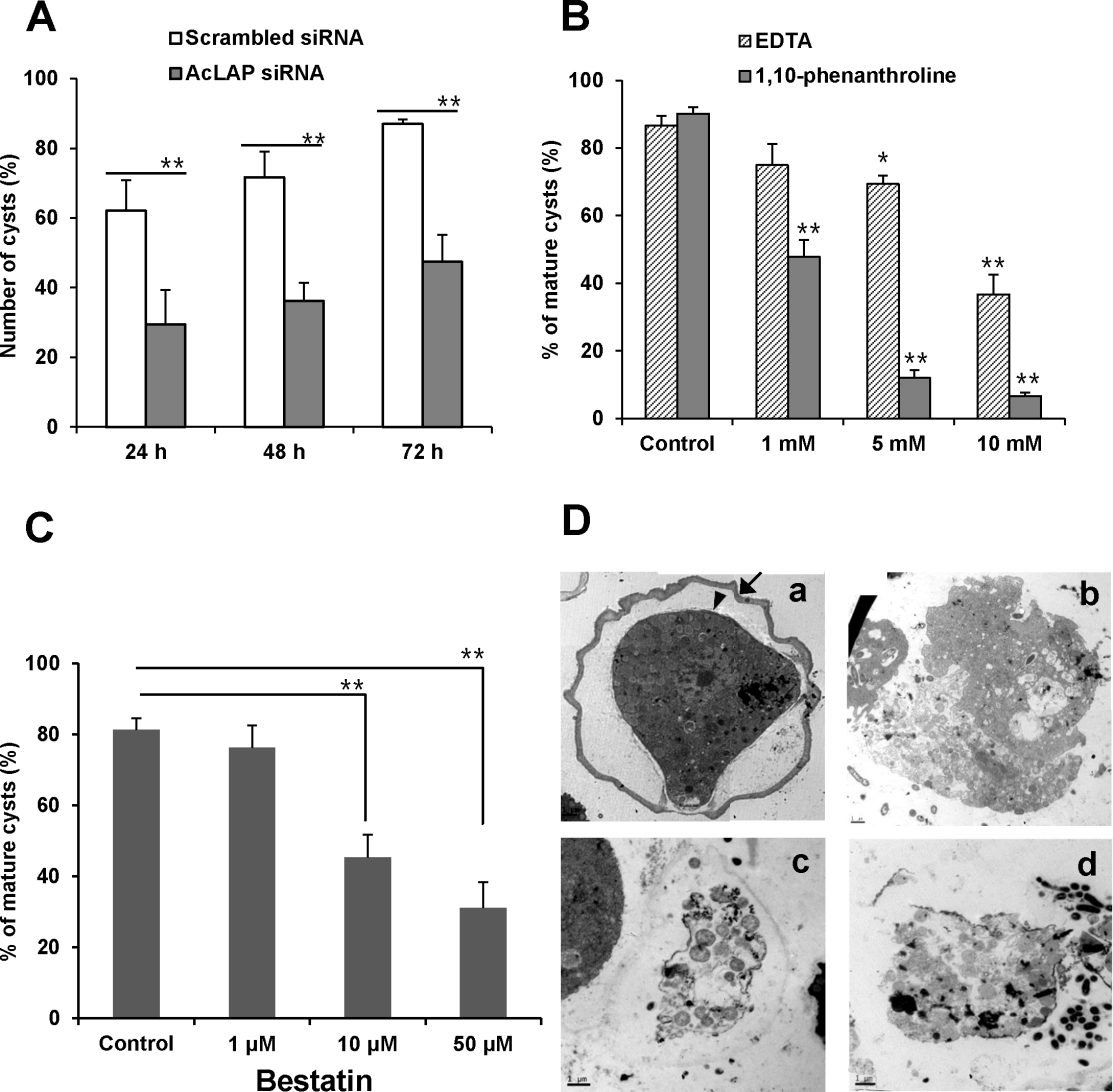
Effects of AcLAP knockdown on encystation. (A) Trophozoites transfected with scrambled siRNA (control, open bars) or AcLAP siRNA (gray bars), incubated in encystation medium for 24 h, 48 h, or 72 h and treated with 0.05% sarkosyl for 30 min. Sarkosyl-resistant mature cysts were stained with 0.22% trypan blue and counted under a microscope. (B) Trophozoites treated with different concentrations (1 mM, 5 mM, or 10 mM) of EDTA or 1,10-phenanthroline and PBS (control), transferred into encystation medium, and incubated for 72 h. Mature cysts were counted after sarkosyl treatment. (C) Trophozoites were transferred into encystation medium containing 1 μM, 10 μM, or 50 μM bestatin or control (distilled water) and incubated for 72 h. Mature cysts were counted after sarkosyl treatment. Data represent the means ± SD of three separate experiments; asterisk, *P* < 0.05; double asterisk, *P* < 0.01. (D) Ultrastructural changes in AcLAP knockdown cells revealing a lack of detectable ectocyst and endocyst. Trophozoites transfected with nonspecific scrambled siRNA (a, b) or AcLAP siRNA (c, d) were transferred into encystation medium (b: containing 50 μM bestatin), incubated for 72 h, and examined by transmission electron microscopy (TEM). The black arrow and arrowhead show the ectocyst and endocyst, respectively, in scrambled siRNA-transfected control cells. Bar = 2 μm.

To ascertain whether the decreased LAP activity caused by AcLAP knockdown affects encystation of *A*. *castellanii*, we treated the cells with sarkosyl, to which cysts are resistant, and counted the numbers of cysts formed. Both scrambled siRNA- and AcLAP siRNA-transfected cells started to transform into cysts in gradually increasing proportions after encystation induction. However, at 72 h post-encystation, 87% of the scrambled siRNA-transfected cells were transformed into cysts, while only 47.4% of the AcLAP siRNA-transfected cells (*P* < 0.01) had successfully converted (Fig 5A).

We hypothesized that if encystation was inhibited by suppression of AcLAP expression in AcLAP knockdown cells, treatment with metal chelators or aminopeptidase inhibitors should also inhibit encystation of *Acanthamoeba*. Therefore, we examined the effects of the metal chelators EDTA and 1,10-phenanthroline and the aminopeptidase inhibitor bestatin on encystation of *Acanthamoeba*. Trophozoites were treated with different concentrations of EDTA, 1,10-phenanthroline, or bestatin, transferred into encystation medium to induce encystation, and the number of cysts was counted. As shown in Fig 5B, mature cysts represented more than 80% of control cells but only 37% of cells (*P* < 0.01) treated with 10 mM EDTA at 72 h after encystation induction. Encystation inhibition was even greater when cells were treated with 1,10-phenanthroline: only 6.6% of cells were mature cysts after treatment with 10 mM 1,10-phenanthroline (*P* < 0.01), compared to 90.2% of control cells. These results indicate that encystation was inhibited by metal chelators in a concentration-dependent manner. In the case of bestatin, mature cysts constituted 81% of control cells, but only 31% of cells (*P* < 0.01) were transformed into cysts at 72 h after encystation induction when treated with 50 μM bestatin (Fig 5C). No significant change in viability of trophozoites was detected when the cells were treated with the same concentration of bestatin for 72 h (data not shown). Collectively, these results indicate that AcLAP may play an important role in the encystation process of *Acanthamoeba*.

### Ultrastructural changes in AcLAP knockdown cells

Ultrastructural changes in AcLAP knockdown cells were examined by TEM (Fig 5D). The mature cyst wall of scrambled siRNA-transfected cells presented as two major well defined layers, a laminar fibrous ectocyst (Fig 5D-a, black arrow), and an endocyst composed of fine fibrils in a granular matrix (Fig 5D-a, black arrowhead), as typically detected in *Acanthamoeba* cysts [5, 30]. Meanwhile, AcLAP siRNA-transfected cells showed no clearly identifiable ectocyst or endocyst, indicating cell rupture or loss of cellular contents (Fig 5D-c and 5D-d). Similar morphological changes were found in cells treated with 50 μM bestatin (Fig 5D-b). These results suggest that the lack of or down-regulated AcLAP activity caused ultrastructural changes of the cell wall of *A*. *castellanii* that may be closely associated with inhibition of cyst formation.

## Discussion

During encystation of protozoan parasites, large numbers of organelles and proteins are degraded by endogenous proteases via autophagy and ubiquitin modification. Autophagy is an evolutionarily conserved lysosomal catabolic machinery in eukaryotes that is a critical response to environmental stress including starvation, cellular development, and differentiation, managing the degradation of redundant organelles and molecules [41]. In *Acanthamoeba*, we previously found that an encystation-mediating serine protease [18] and a cyst-specific cysteine protease [42] were localized in lysosomes or autophagolysosomes and played essential roles in autophagosomal degradation during encystation. Similarly, the ubiquitin-proteasome pathway plays an important role in the selective degradation of misfolded, damaged, or unnecessary proteins [43]. Ubiquitin-labeled proteins are cleaved into polypeptides or oligopeptides in proteasomes [44] and are further degraded to single amino acids in the cytoplasm by tripeptidyl peptidase II [45], LAP [46], and thimet oligopeptidase [47]. In a previous microarray study, we found that the expression levels of ubiquitin and its associated proteins were increased during encystation [22].

AcLAPr showed maximum activity at neutral and slightly alkaline pH and had a broad substrate spectrum, which are typical characteristics of cytosolic LAPs owing to the molecular structure of their active site [25, 48]. Moreover, an AcLAP- EGFP fusion protein was localized in the cytoplasm of trophozoites and cysts, suggesting that AcLAP is involved in the final stages of protein catabolism such as the ubiquitin-proteasome pathway, hydrolyzing oligopeptides into free amino acids in the cytoplasm during encystation of *Acanthamoeba*. Although the expressed AcLAP-EGFP fusion protein of *A*. *castellanii* showed a dispersed pattern in the cytoplasm, multiple small vesicle-like structures of fluorescence, especially in the cysts, were observed. The AcLAP-EGFP expression was apparently discriminative with positive staining of LysoTracker Red. However, AcLAP may form as an aggregate due to its overexpression or as a result of its fusion with EGFP. Thus, further studies are required to determine the exact intracellular localization of endogenous AcLAP during the encystation of *Acanthamoeba*.

In this study, the level of AcLAP transcription in AcLAP siRNA transfectants showed a modest decrease (40% of the level in scrambled siRNA transfectants) (*P* < 0.01) at 72 h after inducing encystation. When the parasite was exposed to AcLAP siRNA, the number of cysts was approximately halved (*P* < 0.01). However, the relative leucine aminopeptidase activity did not exactly correspond to the decreased transcriptional level of AcLAP, indicating that a residual amount of AcLAP activity, due to protein expression at the trophozoite stage, had been retained in the siRNA-treated cells.

Proteases are involved in many cellular processes and are key players in the macromolecular turnover associated with the degradation of cellular components. Recent work on *Giardia* and *Entamoeba* has implicated roles of cysteine and serine proteases in encystation [7, 8, 12–14, 49–51]. Several studies suggested that aminopeptidases play important roles during encystation and differentiation of protozoan parasites. In *Cryptosporidium parvum*, excystation is inhibited by the metalloaminopeptidase inhibitor amastatin or by the metal chelator 1,10-phenanthroline [52]. Two aminopeptidases, LAP [53] and methionine aminopeptidase [54], have been identified in *Toxoplasma*, the latter being involved in the differentiation of *Toxoplasma gondii* tachyzoites to bradyzoites. *L*. *major* pyroglutamyl peptidase I, a cysteine aminopeptidase, is involved in differentiation from the procyclic promastigote to the infective metacyclic promastigote [55]. Furthermore, bestatin treatment inhibited cell growth, cell division, and spore differentiation of *Dictyostelium discoideum* by interacting with puromycin-sensitive aminopeptidase A, which is known to regulate cell division and differentiation in other organisms [56]. In *G*. *lamblia*, bestatin significantly inhibited encystation and abolished the expression of cyst wall protein 1 and cyst wall protein 2 [10], suggesting that *G*. *lamblia* aminopeptidases are involved in cyst wall synthesis.

In *Acanthamoeba*, the mature cyst wall is synthesized in the late stages of encystation (20–24 h) [57] and is composed of an outer layer (ectocyst) and an inner layer (endocyst) separated by a space [30]. Because the endocyst is partially composed of cellulose [58], cyst wall synthesis is considered a potential therapeutic target. In the present study, we found that AcLAP activity was elevated in *A*. *castellanii* during encystation and that knockdown of AcLAP resulted in interruption of the normal encystation process. Moreover, bestatin did not affect the viability of trophozoites, but mature cyst formation was significantly inhibited by the same concentration of bestatin. As shown in Fig 5D, cyst walls were impaired or absent in AcLAP knockdown cells, resulting in cell rupture or loss of cellular contents. These results indicate that AcLAP activity is essential for cyst wall formation in *A*. *castellanii*.

It has long been known that divalent metal ions, particularly Mg^2+^ and Ca^2+^, are required during encystation of *Acanthamoeba* [59, 60]. In *A*. *castellanii*, magnesium chloride inhibited the efflux of ultraviolet-absorbing materials, decreased cell loss by lysis, promoted cellulose synthesis and EDTA-inhibited encystation [16], and reduced excystation efficiency, suggesting that divalent ions may affect electrochemical gradients and signaling cascades for encystation. However, the roles of metal ions, related signaling cascades, and metalloproteases in *Acanthamoeba* encystation remain unclear.

LAP is a metalloexopeptidase that belongs to the M17 enzyme family and requires two metal ions, which are situated within its catalytic site and act as intermediaries between the enzyme and its substrate [24]. In this study, the activity of AcLAPr was enhanced by addition of divalent metal ions (Fig 3C) and was inhibited by the metal chelators EDTA and 1,10-phenanthroline (Table 1). Moreover, these metal chelators also inhibited encystation of *A*. *castellanii* (Fig 5B), indicating that divalent metal ions are important for AcLAP activity essential for encystation, consistent with previous results. Khunkitti et al. [61] reported that the biocidal effectiveness of chlorhexidine diacetate against *Acanthamoeba* cysts was enhanced in combination with EDTA. Although EDTA and 1,10-phenanthroline are unlikely to have direct cysticidal effects on *Acanthamoeba*, they reduced encystation efficiency, suggesting their value as a potential therapeutic target for preventing *Acanthamoeba* cyst formation. While we cannot exclude the possibility that metal chelators affect electrochemical gradients and signaling cascades necessary for successful encystation, as hypothesized in the previous study, we know that metal ions are required in some way for AcLAP activity associated with encystation or cyst wall synthesis in *Acanthamoeba*. In contrast to the membrane-associated dipeptidyl peptidase IV of *Giardia* [10] or the secreted metalloprotease of *Acanthamoeba* [19–21], AcLAP is a cytosolic aminopeptidase. Although the exact function of AcLAP remains unknown, we hypothesize that cyst wall synthesis was blocked by the impaired turnover of proteins via autophagy or the ubiquitin-proteasome pathway, or that cysts were ruptured by the accumulation of undegraded oligopeptides in AcLAP knockdown cells. Bestatin also reduced cyst formation, while this transition-state analog is active against the ubiquitous M1-family aminopeptidase [23]. Along with AcLAP, we recently cloned metalloproteases from *A*. *castellanii* that are inhibited by aminopeptidase inhibitors or metal chelators. Therefore, we cannot exclude the possibility that other metalloproteases, which are affected by metal chelators, are involved in encystation of *A*. *castellanii*. Further studies are required to identify proteases associated with encystation and excystation of *Acanthamoeba* and to evaluate the roles of divalent metal ions in these processes.

## Supporting Information

S1 Fig Expression and purification of AcLAPr and preparation of polyclonal antibody against AcLAPr. (A) SDS-PAGE and Coomassie blue staining of the whole cell lysate corresponding to *E*. *coli* BL21 transformed with pCold-TF-AcLAP plasmid (lane 1), soluble fraction of the sonicated cell lysate (lane 2), eluted AcLAPr from the Ni-NTA agarose column (lane 3), thrombin-cleaved and purified AcLAPr (lane 4), and crude cyst extract at 72 h after induction of encystation of *A*. *castellanii* (lane 5). Protein marker (lane M). (B) The same samples as used for the experiment described in panel A were diluted 100-fold (lane 1–4), except crude cyst extract of *A*. *castellanii* (lane 5). Western blot analyses were carried out with rat polyclonal anti-AcLAPr antibody.
(PDF)
