# Supplementary figures and images for: Essential Role for an M17 Leucine Aminopeptidase in Encystation of Acanthamoeba castellanii
Source: PLoS One. 2015 Jun 15;10(6):e0129884. doi: 10.1371/journal.pone.0129884 (PMC4468156; doi:10.1371/journal.pone.0129884)

**A**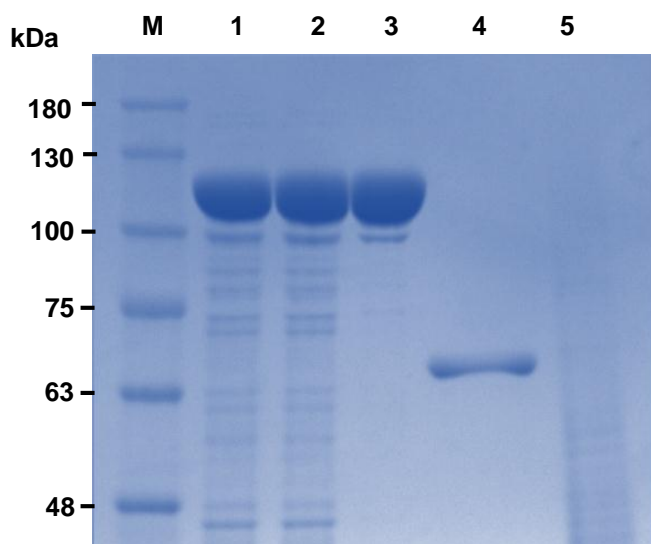**B**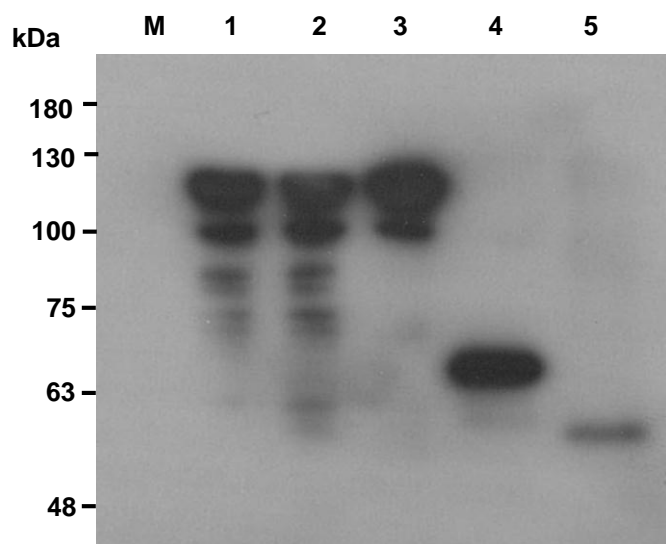

Supplement: S1 Fig — (A) SDS-PAGE and Coomassie blue staining of the whole cell lysate corresponding to E. coli BL21 transformed with pCold-TF-AcLAP plasmid (lane 1), soluble fraction of the sonicated cell lysate (lane 2), eluted AcLAPr from the Ni-NTA agarose column (lane 3), thrombin-cleaved and purified AcLAPr (lane 4), and crude cyst extract at 72 h after induction of encystation of A. castellanii (lane 5). Protein marker (lane M). (B) The same samples as used for the experiment described in panel A were diluted 100-fold (lane 1–4), except crude cyst extract of A. castellanii (lane 5). Western blot analyses were carried out with rat polyclonal anti-AcLAPr antibody. (PDF) [file pone.0129884.s001.pdf]
